# Point-of-Care Ultrasound Training: An Assessment of Interns’ Needs and Barriers to Training

**DOI:** 10.7759/cureus.11209

**Published:** 2020-10-28

**Authors:** Waleed Jarwan, Abdullah A Alshamrani, Afnan Alghamdi, Naveed Mahmood, Yousuf M Kharal, Rajkumar Rajendram, Arif Hussain

**Affiliations:** 1 Internal Medicine, College of Medicine, King Saud Bin Abdulaziz University for Health Sciences, Riyadh, SAU; 2 Internal Medicine, King Abdulaziz Medical City, Riyadh, SAU; 3 Internal Medicine, College of Medicine, Alfaisal University, Riyadh, SAU; 4 Anesthesiology/Cardiac and Critical Care, King Abdulaziz Medical City, Riyadh, SAU

**Keywords:** point-of-care ultrasound, education needs assessment, curriculum development, internship, medicine

## Abstract

Background and objective

The use of point-of-care ultrasound (POCUS) is generally on the rise worldwide. However, as the epidemiology of diseases and the approach to their management vary internationally, POCUS may not be universally applicable. The resources available for medical education are generally limited. Thus, when considering the development of a training program during the internship year, we sought to determine interns' perceptions of the applicability of POCUS to clinical practice, the current skill gaps, and barriers to training.

Methods

A validated questionnaire was distributed to the interns of the College of Medicine, King Saud bin Abdulaziz University for Health Sciences (KSAU-HS), Riyadh to determine their proficiency in POCUS, and their opinions on its applicability on a 5-point Likert scale. Each skill gap was calculated by subtracting self-reported proficiency in POCUS from its perceived applicability.

Results

Of the 300 total interns (male: 200, female: 100), 229 participated [response rate: 76%; male: 136 (68%), female: 93 (93%)]. The use of POCUS to detect abdominal free fluid was perceived to be the most applicable use (mean: 3.9 ±1.1); scanning for consolidation was the least applicable (mean: 3.0 ±1.2). Knowledge and proficiency among the sample were generally poor. The skill gap was greatest for the assessment of inferior vena cava collapsibility (mean: 1.4 ±1.3) and least for the identification of pneumothorax (mean: 0.5 ±1.5). Although three-quarters of the participants (170) agreed that POCUS was an essential skill, 36 (16%) stated that they had no interest in it, and nearly half (101) believed that they did not have time to learn POCUS.

Conclusions

While POCUS is applicable to medical interns in Saudi Arabia, significant skill gaps exist. However, our sample's perception of the applicability of POCUS was less favorable than that of internal medicine (IM) residents in Canada. Thus, initiating POCUS training during the internship year may yield suboptimal results. Interns must prioritize medical licensing examinations and applications for residency training. Indeed, many interns believe that they do not have enough time to learn POCUS. Thus, prioritizing the training of residents in POCUS may be a more effective use of the finite resources available for medical education.

## Introduction

Point of care ultrasound (POCUS) is a fast, portable, and non-invasive diagnostic tool [1-3]. Physicians proficient in its use can quickly answer specific clinical questions at the bedside. The diagnostic accuracy of POCUS for detection of many pathologies (e.g., pleural effusion, pneumothorax, pneumonia, and interstitial syndromes [4-9], hydronephrosis [1,2], hepatomegaly [10], splenomegaly [11], and ascites [10]) is excellent. Therefore, POCUS is an invaluable adjunct to bedside diagnostic evaluation.

There is substantial evidence to support the clinical value of POCUS to several specialties [1-3,12]. Yet, surprisingly, practicing physicians have been mostly reluctant to integrate this paradigm-shifting technology into their routine practice [1]. This is partly due to their lack of familiarity with the tool [1]. As POCUS is a relatively new technology, most frontline physicians have little or no experience with its use. Furthermore, POCUS is highly operator-dependent [4]. To be effective, POCUS must be performed by competent practitioners [4].

Safe, competent, and effective use of POCUS requires training to close gaps in learners’ knowledge and skill [13,14]. Fortunately, recent data suggest that those keen to learn POCUS can obtain adequate proficiency with minimal training [10,15].

While Saudi Arabia does not have any curricula for POCUS training as yet, several national and international bodies have developed specialty-specific POCUS curricula [12,16-21]. Since POCUS is applicable in many different medical areas, learning POCUS is likely to be relevant to the medical interns. However, the integration of POCUS into routine clinical practice requires a substantial investment, and the resources available for medical education are generally limited. The development of a POCUS training program for interns must therefore be justified. A needs assessment is required to confirm that training interns to use POCUS is both required and appropriate [13,22].

The primary aims of this study were therefore to determine interns’ perceptions of the applicability of POCUS to their clinical practice, quantify their self-reported ability to perform POCUS, and thereby define the skill gaps in an internship program in Saudi Arabia. The secondary objective was to determine the barriers to training this cohort in POCUS.

## Materials and methods

Study design

This cross-sectional survey was performed among the interns of the internship program at the College of Medicine, King Saud bin Abdulaziz University for Health Sciences (KSAU-HS), Riyadh, Saudi Arabia.

Questionnaire development

Studies describing the applications of POCUS and the competencies required for its safe practice were reviewed [1,3,10-13,16,23,24]. Drawing on this knowledge, two researchers with expertise in medical education, POCUS, and survey design (RR and AH) developed a validated questionnaire to investigate interns' perceptions of POCUS. The survey instrument was divided into six sections.

The first section requested demographic data (i.e., age and gender). The second section included questions on training, accreditation, and the use of POCUS. The third section investigated the applicability of 15 diagnostic indications for POCUS (i.e., a needs assessment). For each diagnostic application, participants were asked the following question: How applicable is this indication for POCUS to your clinical practice? The fourth section explored participants’ ability to perform POCUS (i.e., self-reported proficiency in each of the 15 indications for POCUS included in section three). The fifth section asked participants to self-report their knowledge of the principles of ultrasound relevant to POCUS. This section included 16 items. The sixth section asked interns to reflect on their attitudes towards training in POCUS and the barriers to training.

After obtaining ethical approval, the survey instrument (Appendix 1) was piloted with three pediatric residents to obtain input on survey length, content, and clarity. It was universally agreed that no changes were required.

Participants

The study was conducted among the interns at the internship program of the College of Medicine, KSAU-HS, Riyadh, Saudi Arabia. During the academic year encompassing the period July 1, 2020, to June 30, 2021, there were 300 interns (male: 200, female: 100) enrolled in this program. Assuming a response distribution of 50%, it was estimated that at least 169 interns (male: 132, female: 80) would have to participate to obtain a 5% margin of error at a level of confidence of 95%. Thus, to allow for some anticipated refusal to participate, all interns being trained at the College of Medicine, KSAU-HS were invited to participate. The questionnaire was distributed to interns via an email link to an online form (Google Forms; Google, LLC, Mountain View, CA) in September 2020. Informed consent was obtained from all participants before the survey. No incentives were provided.

Study outcomes

The training and accreditation in POCUS were determined using close-ended questions (i.e., yes/no). The use of POCUS was assessed using an incremental scale (never, once a month, once a week, daily, more than once a day). A 5-point Likert scale (1: very poor, 2: poor, 3: fair, 4: good, 5: very good) was used to assess the interns’ perceived applicability of POCUS and self-reported proficiency and knowledge. A skill gap was calculated for each participant for each indication of POCUS by subtracting individual intern’s self-reported proficiency in each skill from their perceived applicability of that skill [13]. This method of calculating a skill gap has been described previously [13].

Ethical approval

Ethical approval for this study was obtained from the institutional review board of King Abdullah International Medical Research Center, Riyadh, Saudi Arabia. 

Statistical analysis

The data were analyzed using standard descriptive statistical techniques. The final analysis included all responses. Interns’ responses were stratified by gender. To facilitate the comparison of data, interval data, described as a 5-point Likert scale, were presented as both frequencies and mean ±SD, as described previously [13]. The skill gaps identified in our cohort were compared to those described in a Canadian study [13]. The data were compared using Student’s t-tests or analysis of variance (ANOVA) as appropriate. Categorical variables were compared using McNemar's or Chi-squared tests. All analyses were performed using Microsoft Excel, version 2016 (Microsoft Corporation, Redmond, WA).

## Results

Demographic data and response rates

The response rate was high (76.3%). Of the 300 medical interns (male: 200, female: 100), 229 (male: 136, female: 93; mean age 24.1 ±1.4 years) participated. Female participants’ response rate (93%) was significantly higher than that of men (68%; χ^2^: 25.9, p<0.0001).

Interns’ attitudes towards training in POCUS

Table 1 shows the sample's attitude towards training in POCUS. The majority of the sample either agreed or strongly agreed with statements that POCUS is an essential skill (170, 74%; male: 96), that every department should have an ultrasound machine (172, 75%; male: 96), and lack of access to ultrasound out-of-hours compromises patient care (170, 74%; male: 93). A total of 140 interns (61%; male: 73) agreed or strongly agreed with all three statements. However, approximately 5% of the sample strongly disagreed with each of these statements.

**Table 1:** Interns’ attitudes towards POCUS Data are presented as frequencies and percentages POCUS: point-of-care ultrasound

Statement about point-of-care ultrasound	Likert scale response, n (%)				
	Strongly disagree	Disagree	Neutral	Agree	Strongly agree
Point-of-care ultrasound is an essential skill	14 (6%)	2 (0.9%)	43 (19%)	75 (33%)	95 (41%)
Lack of access to ultrasound out-of-hours compromises care	11 (5%)	4 (2%)	44 (19%)	99 (43%)	71 (31%)
Need an ultrasound machine in every department	10 (4%)	9 (4%)	38 (17%)	75 (33%)	97 (42%)

Applicability of POCUS to interns’ practice in Saudi Arabia

**Figure 1:**
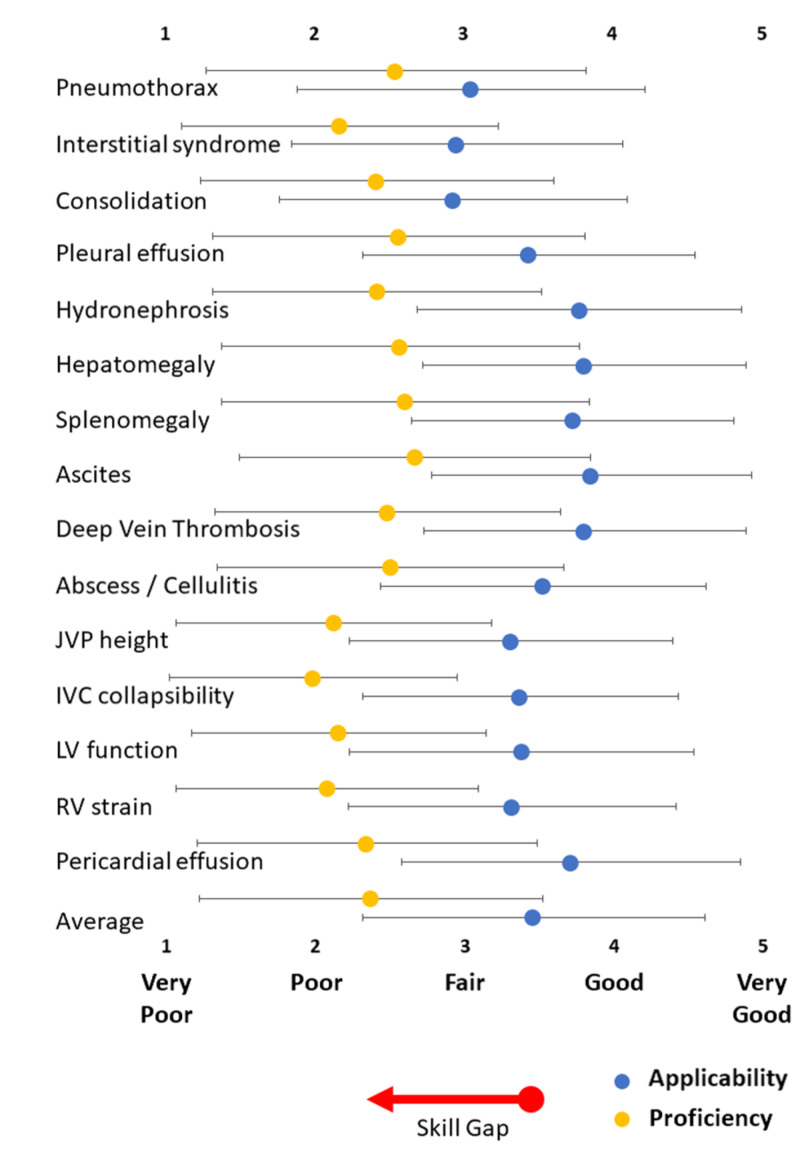
Interns’ perceptions of the applicability of POCUS and their self-reported proficiency in POCUS Applicability and proficiency are rated on a 5-point Likert scale (1: very poor, 2: poor, 3: fair, 4: good, 5: very good). Differences between proficiency and applicability (i.e., the skill gap) for each indication for POCUS were statistically significant (p<0.00001). The red arrow indicates the overall skill gap (i.e. the difference between the average applicability and self-reported proficiency for all indications for POCUS). Data are presented as mean ±standard deviation POCUS: point-of-care ultrasound; DVT: deep vein thrombosis; JVP: jugular venous pressure; IVC: inferior vena cava; LV: left ventricle; RV: right ventricle

The applicability of 15 diagnostic indications for POCUS to interns practicing in Saudi Arabia is shown in Figure 1. There were statistically significant differences between the groups’ means as determined by one-way ANOVA (F(14,3417)=18.7, p<0.00001). The combined applicability of all indications of POCUS was midway between fair and good [mean applicability: 3.5 ±1.1; 2,844 responses (82.8%) were fair, good, or very good; 1,795 responses (52.3%) were good or very good].

Scanning to detect abdominal free fluid was the most applicable use (mean applicability: 3.9 ±1.1). Although the use of POCUS to detect pleural effusion was thought to be applicable (mean applicability: 3.5 ±1.1), the sample perceived that other indications for lung ultrasound were less relevant. Indeed, of the 15 indications for POCUS considered in the present study, scanning for consolidation (mean applicability: 3.0 ±1.2), interstitial syndrome (mean applicability: 3.0 ±1.1), and pneumothorax (mean applicability: 3.1 ±1.2) were perceived to be the least applicable.

A survey of 253 Canadian internal medicine (IM) residents also reported that the use of POCUS to detect abdominal free fluid was the most applicable use (mean applicability: 4.9 ±0.4) [13]. The survey of Canadian IM residents also reported that the applicability of POCUS for the assessment of left ventricular function (mean applicability: 4.8 ±0.5) and pericardial effusion (mean applicability: 4.7 ±0.5) were also very high [13]. However, our sample’s perception of the applicability of these three indications for POCUS was significantly less favorable than that of the Canadian IM residents (p<0.0001).

Interns’ training, accreditation, and use of POCUS

While 51 (22%; male: 20) participants reported that they had received some training in POCUS as undergraduates, only three (1.3%, male: three) had received training in POCUS as interns. Of the 51 participants who had received some training in POCUS, 28 (12%; male: 10) had never used POCUS.

At the other end of the spectrum, some interns reported that they had obtained accreditation in POCUS [16 (8.3%); male: 12] and focused cardiac ultrasound [four (1.7%); male: three]. Many more interns reported that they used POCUS at least once a month [93 (41%); male: 51]. Of this group, 68 (30%; male: 39) reported that they had not received any training in POCUS as medical students or interns. Thus, the majority of interns self-reporting regular use of POCUS was self-taught. So, their familiarity with ultrasound machines and knowledge of image optimization, image acquisition, and interpretation were likely to be insufficient.

Interns’ knowledge of POCUS

**Figure 2:**
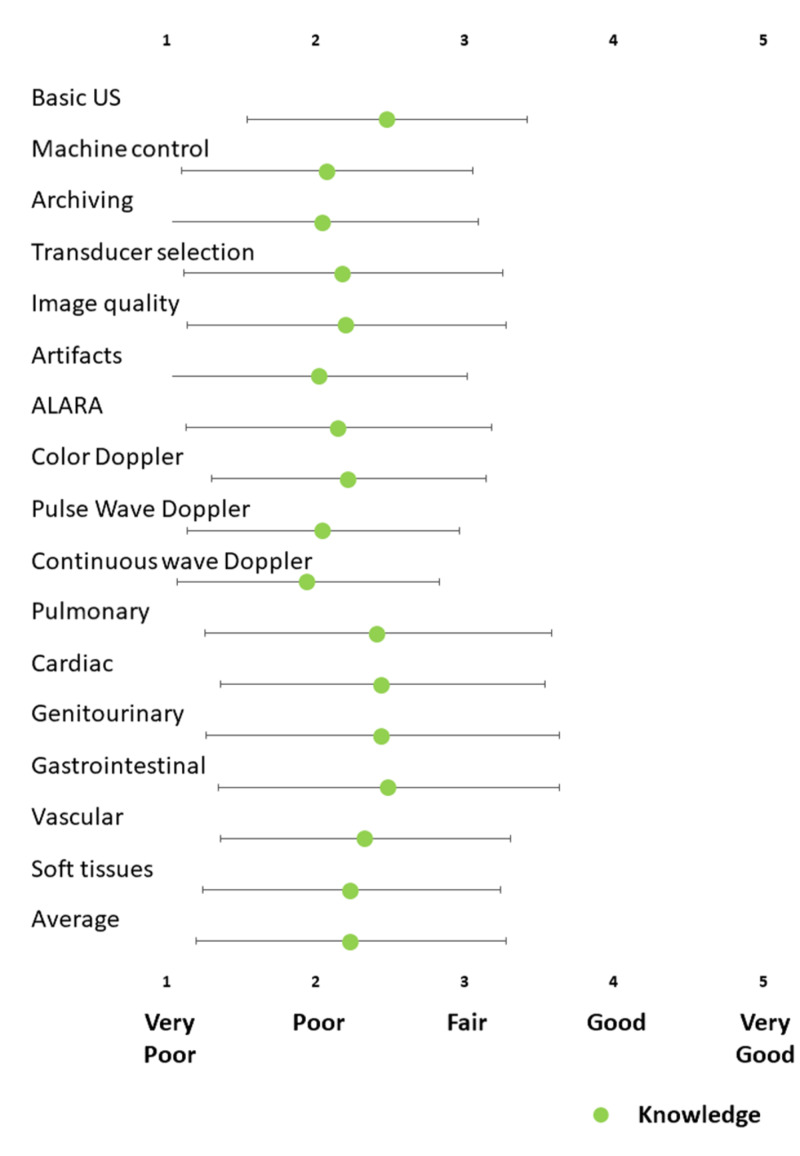
Interns’ knowledge of the principles of ultrasound required to use POCUS Knowledge was self-reported on a 5-point Likert scale (1: very poor, 2: poor, 3: fair, 4: good, 5: very good). Data are presented as mean ±standard deviation POCUS: point-of-care ultrasound; ALARA: as low as reasonably achievable; US: ultrasound

The sample’s overall self-reported knowledge of the principles of ultrasound required to perform POCUS was poor (Figure 2). Self-reported knowledge was greatest in the basic principles of ultrasound (mean: 2.5 ±1.0) and in the interpretation of gastrointestinal findings (mean: 2.5 ±1.2). Participants reported the lowest levels of knowledge regarding pulsed wave (mean: 2.0 ±0.9) and continuous wave (mean: 1.9 ±0.9) spectral Doppler imaging. This lack of knowledge was likely to affect interns’ ability to perform POCUS.

Interns’ proficiency in POCUS and assessment for skill gaps

**Figure 3:**
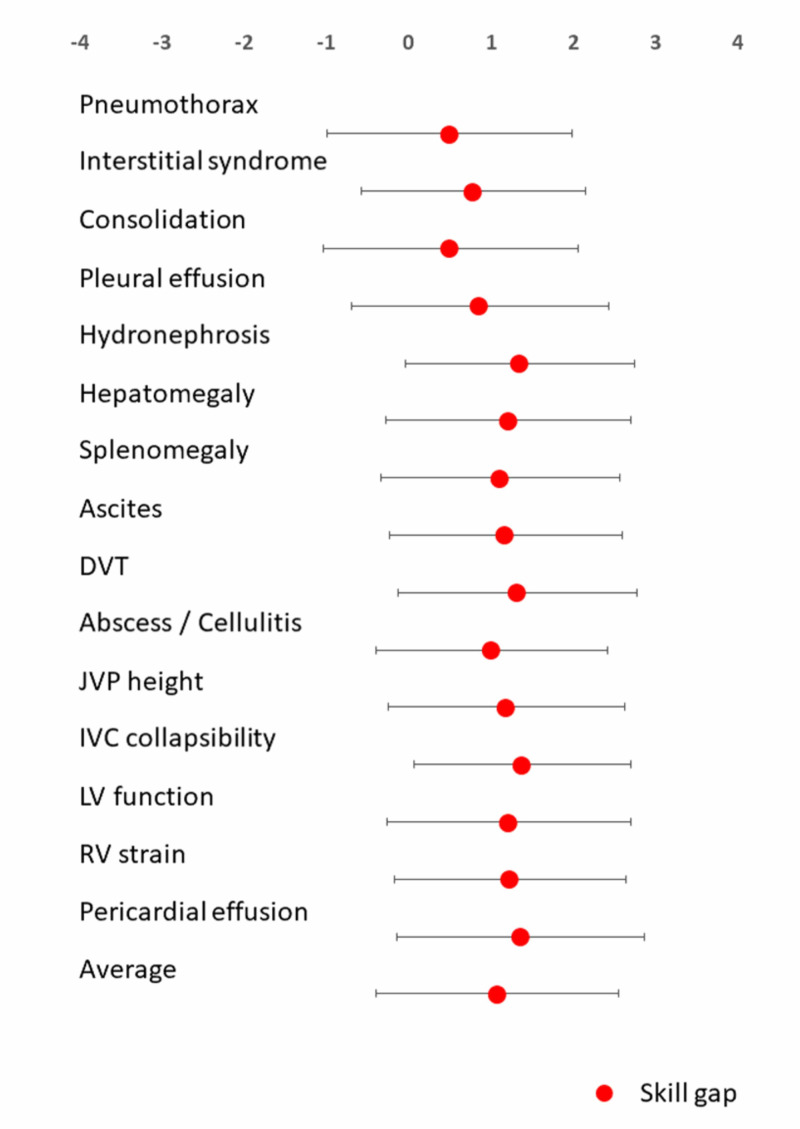
Interns’ skill gaps in POCUS The skill gap for each indication for POCUS was calculated by subtracting individual interns’ self-reported proficiency from their perception of that indications' applicability to patient care. All skill gaps were significant (p<0.00001). Data are presented as mean ±standard deviation POCUS: point-of-care ultrasound; DVT: deep vein thrombosis; JVP: jugular venous pressure; IVC: inferior vena cava; LV: left ventricle; RV: right ventricle

The interns' self-reported proficiency in POCUS is displayed in Figure 1. The sample of interns generally reported poor proficiency in POCUS. The mean of the combined self-reported proficiencies was closer to "poor" than to "fair" (mean proficiency: 2.4 ±1.2). Furthermore, for all the indications studied, the self-reported proficiency in our sample of interns was significantly lower (p<0.0001) compared to their perception of the applicability of POCUS. These observations suggest the presence of skill gaps. The skill gaps are illustrated in Figure 3. The skill gap was greatest for the assessment of inferior vena cava collapsibility (mean: 1.4 ±1.3) and least for the identification of pneumothorax (mean: 0.5 ±1.5).

The Canadian IM residents reported the lowest level of skill in the use of POCUS to detect deep vein thrombosis (DVT; mean skill level: 1.7 ±0.8), hydronephrosis (mean: 1.7 ±0.8), and interstitial syndrome (mean: 1.8 ±0.9) [13]. For comparison, our sample reported the following mean skill levels: DVT mean skill level: 2.5 ±1.2; hydronephrosis mean skill level: 2.5 ±1.1; and interstitial syndrome mean skill level: 2.2 ±1.1.

While the self-reported proficiency in our sample was statistically significantly higher than that of the Canadian IM residents, the differences were small and clinically insignificant. In the survey of Canadian IM residents described previously, skill gaps were highest for identifying DVT (mean gap: 2.7 ±1.1), right ventricular strain (mean gap: 2.7 ±1.1), and gross left ventricular function (mean gap: 2.7 ±1.0). However, our sample’s skill gaps in three indications for POCUS (DVT mean gap: 1.3 ±1.4; right ventricular strain mean gap: 1.2 ±1.4; gross left ventricular function mean gap: 1.2 ±1.5) were significantly less than that of the Canadian IM residents (p<0.0001). This is predominantly due to our sample's perception of POCUS as less applicable than the Canadians perceived it to be.

Barriers to training in POCUS

**Figure 4:**
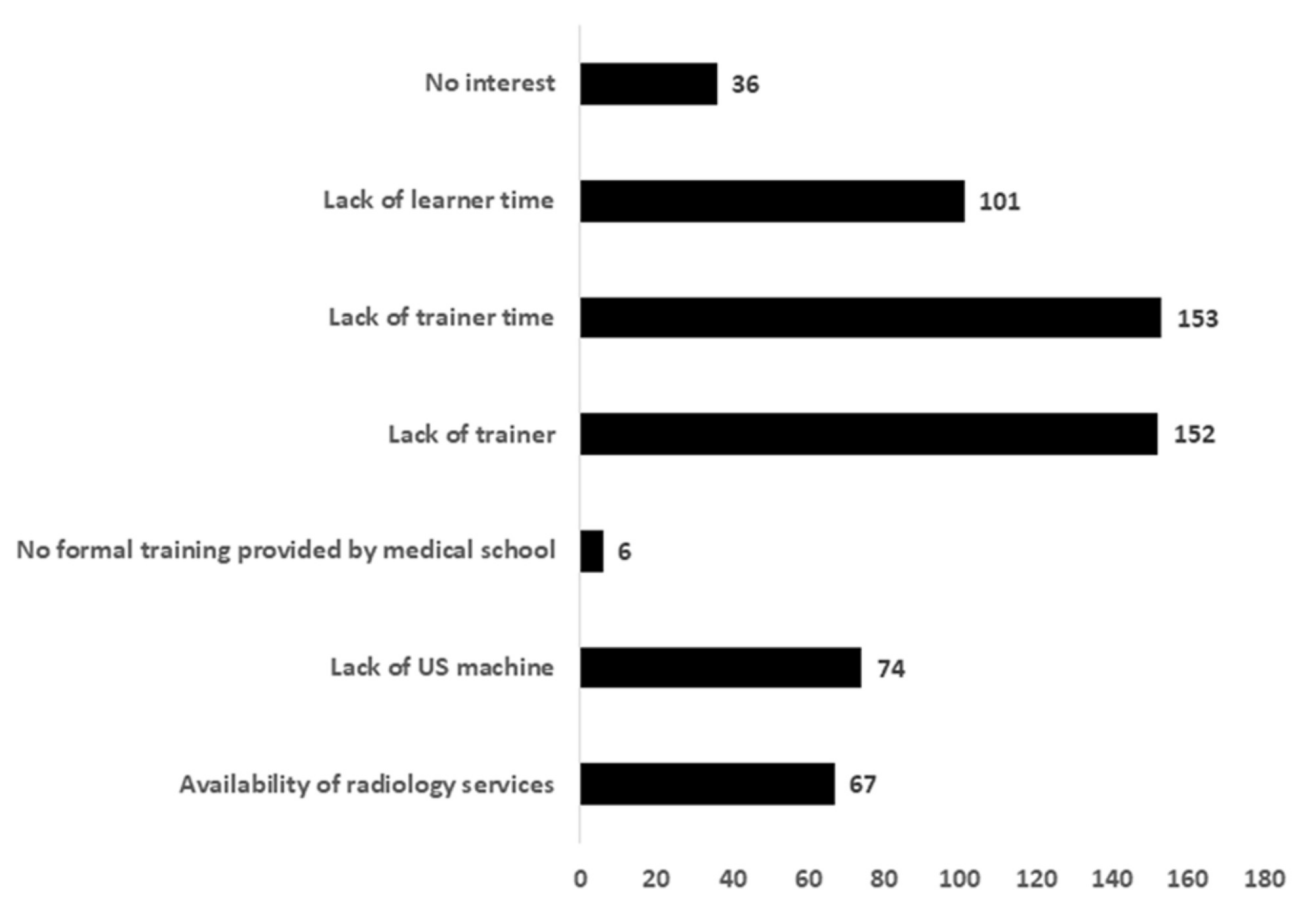
Barriers to learning POCUS during internship training Data are presented as frequencies POCUS: point-of-care ultrasound; US: ultrasound

The barriers to training in POCUS reported by the sample are illustrated in Figure 4. The average number of barriers cited per intern was 2.6. Lack of interest was often cited as a barrier to training (36, 16%; male: 23). Lack of trainers (153, 67%; male: 93) and lack of trainer time for training (156, 67%; male: 101) were the most commonly cited barriers. However, it is important to note that 44% (101, male: 69) reported that lack of learner time was a barrier to learning POCUS.

## Discussion

POCUS is an accurate tool for investigating disease at the bedside [1-3,10,11]. However, the epidemiology of diseases and facilities available within the Middle East vary significantly from those in other regions [25-27]. Medical schools worldwide have integrated POCUS into their curricula [24]. However, there is currently no consensus on the need for POCUS training during the internship year. As the cost of developing and implementing such a curriculum is high, it is important to perform a needs assessment.

Attitude towards learning POCUS, undergraduate training in POCUS, and current use

Three-quarters of our sample of interns believed that POCUS is an essential skill. Yet, only 22% had received any training in POCUS as medical students. Approximately 75% of a similar cohort in the USA reported using POCUS in 2012 and 2013. However, in 2020, significantly fewer interns in Saudi Arabia (41%) use POCUS (χ^2^: 145.9, p<0.00001). Perhaps even more concerning than this data is the finding that most of the interns who reported using POCUS regularly were self-taught. This raises significant concerns for patient safety and clinical governance.

POCUS is a relatively new discipline, and it has not yet been included in the undergraduate medical curricula in Saudi Arabia. It is therefore not surprising that training in sonography and the use of POCUS varied greatly among the interns in our sample. To justify the high investment required to develop a standardized POCUS training program during the internship, it is important to confirm whether the interns in Saudi Arabia require and desire this skill. The current study, therefore, describes interns’ perception of the applicability of 15 indications for POCUS to patient care.

Interns’ perceptions of the applicability of POCUS

The sample reported that POCUS is applicable to their practice (Figure 1). Scanning for abdominal free fluid was perceived to be the most applicable use to their clinical practice (mean applicability: 3.9 ±1.1). This is probably because this skill is applicable to both medical and surgical specialties. However, our sample perceived that this skill was less relevant than a cohort of Canadian IM residents considered it to be.

Although our study was performed at the tail end of the coronavirus disease 2019 (COVID-19) pandemic in Saudi Arabia, the sample perceived that the use of POCUS to detect consolidation and interstitial syndrome were the least applicable use to their clinical practice. This may be because the applicability of lung ultrasound is perceived to be predominantly limited to pulmonologists. However, the sample generally self-reported poor knowledge of ultrasound principles (Figure 2) and poor ability to perform POCUS (Figure 1). So, this failure to recognize the value of lung ultrasound may reflect a lack of awareness about the recent literature that highlights the role of lung ultrasound in the management of COVID-19 [28].

Interns’ self-reported ability to perform POCUS

Our sample’s proficiency in POCUS was generally poor. The difference between self-reported ability to perform a skill and the perceived usefulness of that skill can be used to measure a skill gap [13]. The assessment of the skill gaps (Figure 3) can guide educational interventions to resolve these deficiencies.

Evaluation of the skill gaps

The scores relating to the applicability of each indication of POCUS studied was higher than that of the sample’s self-reported proficiency in that skill (Figure 1). These observations suggest the presence of significant skill gaps relating to POCUS (Figure 2). These gaps can only be addressed by the institution of a training program with formal processes for supervision, governance, and accreditation.

However, the skill gaps were less than those identified in a survey of Canadian IM residents. This difference was predominantly because our sample of interns perceived that POCUS was less relevant than the Canadian IM residents did. This suggests that our sample may have less interest in learning POCUS. Indeed, 16% stated that they had no interest in learning POCUS, and several other barriers to learning POCUS were frequently described (Figure 4).

How can the barriers to learning POCUS be overcome?

To overcome the most frequently cited barriers, the availability of trainers must be increased. The number of ultrasound machines must also be increased. While lower-cost handheld devices are available, ultrasound machines can cost up to 400,000 Saudi Riyal (SAR) depending on size and quality. Thus, overcoming these barriers will require a significant financial investment. Unfortunately, approximately 45% of the sample stated that they did not have sufficient time to learn POCUS. This barrier will be challenging to overcome. To the uninitiated, learning any new skill seems daunting at the outset. However, those keen to learn POCUS can become proficient with minimal training [10,15].

Regardless of these concerns, there are multiple competing demands on interns’ time that must be prioritized for their immediate career progression. For example, interns must sit and pass the Saudi Medical Licensing Examination and then apply for residency training. Thereafter, they must learn the practical skills (including POCUS) required to deliver patient care in their chosen specialties. While it may be most appropriate to initiate training during medical school, until this is realized, our data suggest that it is best to defer training in POCUS until residency programs. Instructing trainees in the most relevant applications of POCUS to their chosen specialty is probably the most efficient use of the resources currently available for medical education.

Strengths and limitations

The study was conducted at the beginning of the academic year, and at a time when the interns had only completed approximately three months of training and were preparing for the Saudi Medical Licensing Examination. Our observations and recommendations are, therefore, also likely to be relevant to medical students immediately prior to the completion of their undergraduate training. Some interns may increase their proficiency in POCUS during their internship. However, as nearly half of our sample believed that they do not have sufficient time to learn POCUS and given that interns at KSAU-HS do not receive formal instruction in POCUS during their internship, our observations are also likely to reflect the ability of junior residents.

While the response rate to the survey was very high, the study has some limitations. Our data involve self-reported knowledge. There are many potential causes of bias in such data [29]. However, interns’ self-reported proficiency in POCUS was generally poor. This is consistent with our personal observations.

Our study was conducted among the interns at the internship program of only one college of medicine in Riyadh, Saudi Arabia. So, its generalizability may be limited. However, KSAU-HS has a large internship program. Our participants’ views are therefore likely to represent those of interns training throughout Saudi Arabia and indeed other countries in which the internship year is punctuated by medical licensing examinations and applications for residency training. Our observations and the views of our sample should therefore be taken into account when developing training programs to safely and effectively integrate POCUS into physicians' training.

Contribution to the existing literature

The presented data provide robust evidence that POCUS is perceived to be an important and applicable tool by the majority of medical interns training in Saudi Arabia. However, currently, training in POCUS at medical school is inconsistent and is almost non-existent during internship training. As a result, our sample’s knowledge and ability to perform POCUS varied but was poor overall. This is likely to be true throughout Saudi Arabia. So, our data suggest that interns training in Saudi Arabia have significant skill gaps in POCUS. However, many interns believe that they do not have sufficient time to learn POCUS during their internship, and some are simply not interested.

Therefore, rather than advocating for the formal inclusion of training in POCUS during the internship, we recommend that POCUS is fully integrated into undergraduate curricula instead. Indeed, several medical schools have already implemented this [24]. However, for the foreseeable future, most physicians will have to resolve this deficiency during residency or fellowship training. It is therefore important for residency and fellowship program directors to be aware of these skill gaps.

## Conclusions

Our data suggest that although interns training in Saudi Arabia perceive that POCUS is applicable to patient care, their exposure is variable, and their proficiency is poor overall. However, the perceived applicability and skill gaps identified in our study were less than those reported in a study of Canadian IM residents. Furthermore, nearly half of the sample believed that they did not have time to learn POCUS. Thus, the internship year may not be the most appropriate time to initiate the training required to resolve these skill gaps. Interns must focus on medical licensing examinations and applications for residency training programs.

The initiation of training in POCUS during medical school would be the ideal solution. However, it will take many years to reap the benefits of developing and implementing such a curriculum for medical undergraduates. Regardless, at least until this is achieved, the skill gaps identified in our study will have to be resolved during residency or fellowship training. Our observations can guide the development of a program that satisfies the perceived needs of clinicians. Those responsible for undergraduate and postgraduate training programs should consider our findings when developing curricula for POCUS.

## Appendices

Study of the applicability of point-of-care ultrasound (POCUS) to interns in Saudi Arabia: Survey of interns’ training, experience, accreditation, and knowledge of POCUS

1. DEMOGRAPHIC DATA

Age 

**Gender: **Male  Female

2. ULTRASOUND EXPERIENCE AND TRAINING

Did you receive/are you receiving formal training in POCUS during medical school?

No                YES             Approximate Total Number of Days of training

Did you receive/are you receiving formal training in POCUS during your internship?

No                YES             Approximate Total Number of Days of training

Do you have any formal accreditation in POCUS?

No                YES              If Yes - what

Do you have any formal accreditation in Echocardiography?

No                YES              If Yes - what

How often do you use POCUS in clinical practice?

Never     Once per month      Once per week        3-4x per week         Daily            > Once/day

3. INTEREST IN LEARNING & 4. CURRENT PROFICIENCY

How applicable to your clinical practice are the following indications for POCUS?

Please describe your proficiency in the following indications for POCUS.

To do this please rate the questions in Table 2 on a 5 point scale

 1                     2                   3                  4                   5

Very poor      Poor             Fair              Good            Very Good

**Table 2:** Questions on the applicability of POCUS to interns' clinical practice and their current level of skill/knowledge POCUS: point-of-care ultrasound; DVT: deep vein thrombosis; JVP: jugular venous pressure; IVC: inferior vena cava; LV: left ventricular; RV: right ventricular

Indication for POCUS	Applicability to your clinical practice?	Your current skills/knowledge?
Identifying pneumothorax	1 2 3 4 5	1 2 3 4 5
Identifying interstitial syndrome	1 2 3 4 5	1 2 3 4 5
Identifying lung consolidation	1 2 3 4 5	1 2 3 4 5
Identifying pleural effusion	1 2 3 4 5	1 2 3 4 5
Identifying hydronephrosis	1 2 3 4 5	1 2 3 4 5
Identifying hepatomegaly	1 2 3 4 5	1 2 3 4 5
Identifying splenomegaly	1 2 3 4 5	1 2 3 4 5
Identifying ascites/free fluid	1 2 3 4 5	1 2 3 4 5
Identifying DVT	1 2 3 4 5	1 2 3 4 5
Identifying abscess/cellulitis	1 2 3 4 5	1 2 3 4 5
Determining the JVP	1 2 3 4 5	1 2 3 4 5
Measuring IVC diameter/collapsibility index	1 2 3 4 5	1 2 3 4 5
Identifying gross LV function	1 2 3 4 5	1 2 3 4 5
Identifying RV strain	1 2 3 4 5	1 2 3 4 5
Identifying pericardial effusion	1 2 3 4 5	1 2 3 4 5

5. KNOWLEDGE

The following section seeks your general knowledge and ultrasound skills.

Please rate your own knowledge/skills in the domains in Table 3 on a 5-point scale?

 1                     2                   3                  4                   5

Very poor      Poor             Fair              Good            Very Good

**Table 3:** Questions on knowledge of the principles of ultrasound imaging required to perform POCUS POCUS: point-of-care ultrasound

Knowledge item/skill	Your current level of knowledge/skill
Basic ultrasound knowledge and use	1 2 3 4 5
Knowing how to control the machine	1 2 3 4 5
Ability to archive images or cine-clips	1 2 3 4 5
Transducer selection	1 2 3 4 5
Ability to discern when image is insufficient/inadequate	1 2 3 4 5
Ultrasound artifacts	1 2 3 4 5
The ALARA (as low as reasonably achievable) principle	1 2 3 4 5
Colour Doppler imaging	1 2 3 4 5
Spectral Doppler imaging - pulsed wave	1 2 3 4 5
Spectral Doppler imaging - continuous wave	1 2 3 4 5
Power Doppler imaging	1 2 3 4 5
Ability to interpret findings - cardiac system	1 2 3 4 5
Ability to interpret findings - pulmonary system	1 2 3 4 5
Ability to interpret findings - gastrointestinal system	1 2 3 4 5
Ability to interpret findings - genitourinary system	1 2 3 4 5
Ability to interpret findings - soft tissues	1 2 3 4 5
Ability to interpret findings - vascular/deep vein thrombosis	1 2 3 4 5

6. ATTITUDE TOWARDS POCUS

How much do you agree that POCUS is an essential skill for your specialty?

Strongly Agree        Agree           Neutral         Disagree       Strongly Disagree

How much do you agree that a lack of access to ultrasound services out of hours (whether radiology-led or physician-led) may compromise patient care?

Strongly Agree        Agree           Neutral         Disagree       Strongly Disagree

How much do you agree that every department should have a dedicated ultrasound machine?

Strongly Agree        Agree           Neutral         Disagree       Strongly Disagree

Perceived barriers to training

Please check all that apply

None                                         

No interest                                 

Lack of learner time                    

Lack of supervisor/trainer time

Lack of supervisor/trainer                

No formal training provided by the College of Medicine

Lack of ultrasound machine

Availability of radiology service

Other - please specify

Do you have any other comments or concerns?
